# The Endocannabinoid System Contributes to Electroacupuncture Analgesia

**DOI:** 10.3389/fnins.2020.594219

**Published:** 2021-02-18

**Authors:** Iona J. MacDonald, Yi-Hung Chen

**Affiliations:** ^1^Graduate Institute of Acupuncture Science, China Medical University, Taichung, Taiwan; ^2^Chinese Medicine Research Center, China Medical University, Taichung, Taiwan; ^3^Department of Photonics and Communication Engineering, Asia University, Taichung, Taiwan

**Keywords:** cannabinoid receptors, electroacupuncture, endocannabinoid system, analgesia, pain

## Abstract

The extensive involvement of the endocannabinoid system (ECS) in vital physiological and cognitive processes of the human body has inspired many investigations into the role of the ECS and drugs, and therapies that target this system and its receptors. Activation of cannabinoid receptors 1 and 2 (CB_1_ and CB_2_) by cannabinoid treatments, including synthetic cannabinoids, alleviates behavioral responses to inflammatory and neuropathic pain. An increasing body of scientific evidence details how electroacupuncture (EA) treatments achieve effective analgesia and reduce inflammation by modulating cannabinoid signaling, without the adverse effects resulting from synthetic cannabinoid administration. CB_1_ receptors in the ventrolateral area of the periaqueductal gray are critically important for the mechanisms of the EA antinociceptive effect, while peripheral CB_2_ receptors are related to the anti-inflammatory effects of EA. This review explores the evidence detailing the endocannabinoid mechanisms involved in EA antinociception.

## Introduction

Since its discovery in the 1990s, the complex signaling network of the endocannabinoid system (ECS) has increasingly been seen to be a key player in the regulation of numerous vital physiological and cognitive processes, such as female reproductive events, pain sensation, mood, and in mediating the pharmacological effects of cannabis (Wang et al., 2006; McPartland et al., 2007; Aizpurua-Olaizola et al., 2017; Toth et al., 2019).

The ECS contains two major G-protein-coupled type 1 (CB_1_) and type 2 (CB_2_) cannabinoid receptors that are activated by the psychoactive ingredient of cannabis, Δ^9^-tetrahydrocannabinol (THC). The main basic mechanisms triggered by CB_1_ and CB_2_ receptors are mediated by G proteins that are mostly of the G_i/o_ type, resulting in inhibition of activity of adenylate cyclases, the cAMP cascade and voltage-gated calcium (Ca^2+^) channels, and stimulation of mitogen-activated protein kinase (MAPK) activity (Di Marzo and Petrocellis, 2006). CB_1_ receptors also inhibit voltage-activated Ca^2+^ channels, stimulate inwardly rectifying potassium (K^+^) currents and activate both phospholipase C (through G protein βγ subunits) and PI-3-kinase (Di Marzo and Petrocellis, 2006).

CB_1_ is mainly expressed in the cerebral cortex, basal ganglia, cerebellum, and hippocampus; lower levels of CB_1_ expression are found in the peripheral and autonomic nervous system, as well as the heart, lung, thymus, spleen, and reproductive system (Fulmer and Thewke, 2018). CB_1_ is also expressed in immune cells, at levels of up to 100-fold lower than those of CB_2_ (Fulmer and Thewke, 2018). In the central nervous system (CNS), activation of CB_1_ inhibits excitatory and inhibitory neurotransmission, and the modulation of cognitive, memory, and motor functions, as well as analgesia (Chiurchiu et al., 2015).

CB_2_ is expressed predominantly by cells of hematopoietic origin in the peripheral immune system (in bones, spleen, and skin); low levels of CB_2_ expression are found in other cell types, including epithelial cells, osteogenic cells, cardiomyocytes, fibroblasts, and vascular smooth muscle cells (Fulmer and Thewke, 2018). Evidence of CB_2_ expression in the CNS is controversial and requires further confirmation (Fulmer and Thewke, 2018). Upregulation of CB_2_ is implicated in chronic inflammation of the nervous system, as well as with several cardiovascular and bone disorders (Chiurchiu et al., 2015).

Two endogenous lipophilic molecules, anandamide (AEA, *N*-arachidonoylethanolamide) and 2-arachidonoylglycerol (2-AG), are capable of activating CB_1_ and CB_2_, and are considered to be the main endocannabinoids (Aizpurua-Olaizola et al., 2017; Maccarrone, 2017). These endocannabinoids, as well as various enzymes involved in the biosynthesis and/or degradation of endogenous lipid ligands, comprise the ECS, a complex enzyme and transporter apparatus that affects virtually all central and peripheral systems in mammals (Aizpurua-Olaizola et al., 2017; Toth et al., 2019). With ongoing research identifying more bioactive lipids with cannabimimetic properties, it is reasonable to expect that investigations will gradually offer more insights into the ECS (Aizpurua-Olaizola et al., 2017; Maccarrone, 2017; Toth et al., 2019).

## Targeting the Endocannabinoid System (ECS) for Treating Pain and Inflammation

The involvement of the ECS in several physiological regulation pathways makes it an attractive target for drugs and therapies in the management of pain and inflammation. Activation of endocannabinoids alleviates behavioral responses to acute, inflammatory and neuropathic pain (Palazzo et al., 2010). The CB_1_ receptor is found in high densities in the superficial layers of the spinal dorsal horn (SDH), the dorsal root ganglia, the descending pathway of pain modulation, and the peripheral terminals of primary afferent neurons (Palazzo et al., 2010). The cellular location of CB_1_ receptors is predominantly the presynaptic axon terminals of both γ-aminobutyric acid (GABA)ergic, and glutamatergic neurons (Campolongo and Fattore, 2015). Smaller quantities of CB_1_ receptors are expressed in astrocytes, oligodendrocytes, and microglia, where the receptors regulate synaptic transmission (Zou and Kumar, 2018). CB_2_ receptors predominantly reside in the periphery and represent a target in inflammatory pain processing (Palazzo et al., 2010).

AEA and 2-AG are synthesized on demand and function as retrograde messengers after their release from postsynaptic neurons in areas related to the descending pain modulatory pathway, from where they are transported to the CNS and peripheral terminals of primary afferent neurons, inhibiting neurotransmitter release from presynaptic terminals (Gondim et al., 2012; Kano, 2014). By activating CB_1_ receptors in astrocytes, 2-AG triggers the release of glutamate, which activates the *N*-methyl-D-aspartate receptor (NMDAR) on pyramidal neurons (Zou and Kumar, 2018). Dense populations of endocannabinoid signaling molecules surround synapses in various brain regions including the periaqueductal gray (PAG) region, the hippocampus, cerebral cortex, amygdala, dorsal and ventral striatum, hypothalamus, cerebellum, and spinal cord, all of which are considered to be responsible for the neural functions that depend upon endocannabinoid signaling (Kano, 2014). Experimental data show that retrograde endocannabinoid signaling governs various aspects of neural signaling including learning and memory, anxiety and mood, addiction, feeding behavior, motor learning, and analgesia (Kano, 2014). Activation of CB_1_ and CB_2_ receptors inhibits established inflammatory hypersensitivity and swelling in animal models of inflammatory hyperalgesia (Gondim et al., 2012). Human samples of osteoarthritis and rheumatoid arthritis synovium tissue contain CB_1_ and CB_2_ receptors as well as AEA and 2-AG, indicating that the ECS may be closely related to pain and inflammation associated with arthritic disease (Gondim et al., 2012).

Cannabinoid treatments have proven efficacy in chronic pain and symptom control in palliative/supportive care. These compounds include the plant-derived cannabinoid tetrahydrocannabinol/cannabidiol (THC/CBD) oromucosal spray (nabiximols), and the synthetic THC analogs nabilone, dronabinol, and ajulemic acid, as well as the CBD oral solution Epidiolex^®^, all of which achieve their biological effects by activating cannabinoid receptors. However, while synthetic cannabinoids are generally devoid of many of the side effects of opiates, such as their large abuse potential and the life-threatening side effect of respiratory depression, use of synthetic cannabinoids has been linked to severe illness, intensive care admission, and death (Kasper et al., 2015). Synthetic cannabinoids can also affect the cardiovascular system, with case reports describing adverse outcomes including cardiogenic shock, acute respiratory depression and cardiopulmonary resuscitation (Khan S. P. et al., 2019).

The ECS maintains bodily homeostasis by influencing physiological processes such as cannabinoid signaling in the skin (Toth et al., 2019), emotional stasis (Stampanoni Bassi et al., 2018), immune homeostasis in the gut (Acharya et al., 2017), and the regulation of appetite, food intake, and energy balance (Pagotto et al., 2006), all of which depend on CB_1_ and CB_2_ receptors, among others (Maccarrone, 2017). The present review provides a brief general overview of acupuncture and electroacupuncture (EA) and highlights the therapeutic potential in EA-induced modulation of cannabinoid signaling for effective analgesia and reductions in inflammation. Importantly, these therapeutic outcomes are produced without the adverse effects associated with synthetic cannabinoids.

## Manual Acupuncture and Electroacupuncture Analgesia

Traditional manual acupuncture has a long history, whereas EA was introduced into clinical practice as recently as the 1950s (Napadow et al., 2005; White et al., 2008). During manual acupuncture, needles are inserted into historically and empirically defined acupoint locations and are then manually manipulated by twisting, lifting or thrusting movements (Napadow et al., 2005). The EA technique involves the insertion of two needles that serve as electrodes for passing an electric current (Napadow et al., 2005). At least one of the needles is positioned at an acupoint (Napadow et al., 2005).

Both manual acupuncture and EA have demonstrated clinically relevant effects in chronic pain conditions (Scharf et al., 2006; Endres et al., 2007; Haake et al., 2007; Selfe and Taylor, 2008; Chen et al., 2017). The use of acupuncture within TCM is supported by a wealth of data demonstrating its therapeutic effects in numerous clinical conditions, including pain, such as the treatment of general chronic pain (Vickers et al., 2012), migraine prophylaxis (Linde et al., 2016; Musil et al., 2018) and treatment (Zhao et al., 2005), the treatment of chronic low back pain (Yuan et al., 2008; Chou et al., 2017), fibromyalgia (Deare et al., 2013; Mist and Jones, 2018), and osteoarthritis (Manheimer et al., 2010; Lin et al., 2016). An individual patient data meta-analysis that used data from 29 randomized controlled trials (RCTs) including a total of 17,922 patients found that acupuncture (whether it was manual acupuncture or EA) was significantly superior to both sham and no acupuncture control for all four chronic pain conditions investigated (back and neck pain, shoulder pain, chronic headache, and osteoarthritis) for the efficacy of acupuncture (Vickers et al., 2012). These findings were confirmed in a subsequent update of this meta-analysis (Vickers et al., 2018). The updated review included an additional 13 RCTs of acupuncture (manual acupuncture or EA) compared with either sham acupuncture or no acupuncture control for non-specific musculoskeletal pain, shoulder pain, chronic headache, or osteoarthritis; data were analyzed from a total of 20,827 patients (Vickers et al., 2018).

Manual acupuncture is associated with the activation of all types of afferent fibers (Aβ, Aδ, and C), while the EA current has to be sufficiently intense to excite Aβ-type and some of the Aδ-type afferent fibers for eliciting analgesia (Zhao, 2008). Adding manipulation to EA reportedly produces more potent analgesia compared with EA alone (Kim et al., 2000). An oft-stated advantage of EA for clinical practice or research is that it can objectively and quantifiably define stimulus frequency and intensity (Napadow et al., 2005). Functional magnetic resonance imaging (fMRI) investigations have revealed that EA (2 and 100 Hz) is associated with more widespread increases in fMRI signaling compared with manual acupuncture, while all acupuncture needling is associated with more widespread responses in the brain compared with the placebo-like tactile control stimulation (Napadow et al., 2005). Differential brain activation is observed between manual acupuncture and EA, which has also been noted by other fMRI and positron emission tomography (PET) investigations into acupuncture analgesia in the human brain, suggesting that different brain networks or different brain mechanisms are involved during manual acupuncture and EA (Kong et al., 2002; Napadow et al., 2005; Yang J. et al., 2012).

Electroacupuncture (EA) is used by traditional Chinese medicine (TCM) practitioners and acupuncturists for many different therapeutic conditions (Sidhu et al., 2017). Some acupuncturists contend that manual needling is sufficient to achieve the desired therapeutic results, whereas others argue that EA provides a unique role, especially in patients with chronic nociceptive pain (White et al., 2008). Some research suggests that EA may provide more effective pain relief than manual acupuncture, with the addition of the electric current optimizing the effects elicited by manual acupuncture (Wan et al., 2001; Barlas et al., 2006). Furthermore, low-frequency, high-intensity EA has been associated with a significantly larger hypoalgesic effect compared with placebo needling, whereas low-intensity stimulation was not significantly different from placebo needling in hypoalgesic responses (Barlas et al., 2006).

## The Descending Pain Modulatory Pathway in Acupuncture Analgesia

The pathophysiology of pain has been thoroughly researched by many in-depth papers and reviews (Loeser and Melzack, 1999; Raffaeli and Arnaudo, 2017; Chen et al., 2020). Among the brain and spinal cord areas, the descending pain modulatory pathway is critical in pain perception and acupuncture analgesia (Lv et al., 2019). This system arises in the PAG, where transmitters are activated that contact the rostroventromedial medulla and project to the raphe nuclei in the brainstem, and to inhibitory synapses in the SDH (Lai et al., 2019; Lv et al., 2019). Local administration of GABA agonists into the ventrolateral area of the PAG (vlPAG) promotes pain, while local administration of GABA antagonists produces antinociceptive effects by reducing inhibitory neurotransmission (Zhu et al., 2019). The GABA disinhibition hypothesis proposes that tonically active GABAergic interneurons within the PAG release GABA, which acts via GABA_A_ receptors to inhibit spinally projecting output neurons (Zhu et al., 2019). According to this hypothesis, opioids and cannabinoids indirectly suppress the inhibitory influence of local GABAergic interneurons and effectively disinhibit the antinociceptive pathway of the neuronal output descending to the spinal cord (Zhu et al., 2019). Thus, cannabinoids are capable of producing analgesia via a central action in the descending pain modulatory pathway, direct spinal action, or peripheral nerve action (Gondim et al., 2012).

## Explorations Into Acupuncture- and Electroacupuncture (EA)-Induced Analgesia

### Endogenous Opioid Involvement

In 1973, Chinese investigators performed a clinical study involving healthy volunteers that demonstrated time-dependent analgesic effects of manual acupuncture at acupoint LI4 (Research Group of Acupuncture Anesthesia, 1973). The increase in pain threshold after manual acupuncture at LI4 was blocked by pretreatment with local anesthetic 2% procaine injected deep below LI4, indicating the importance of nerve innervations embedded in structures deep below the acupoint (Research Group of Acupuncture Anesthesia, 1973). When they then treated the affected limbs of hemiplegic and paraplegic patients with acupuncture, the researchers found no effect on pain threshold on the unaffected side, supporting the involvement of peripheral sensory nerves and the afferent nerve pathway in the spinal cord (Research Group of Acupuncture Anesthesia, 1973). The significance of pain relief achieved with the use of LI4 has been highlighted by recent investigations showing immediate, significant relief from inflammatory pain in mice injected with complete Freund’s adjuvant (CFA) in the hind paws (Yen et al., 2019).

In the late 1970s, researchers discovered that the opiate receptor antagonist naloxone attenuated acupuncture analgesia in humans (Mayer et al., 1977) and in mice (Pomeranz and Chiu, 1976). This analgesic action was attributed to the release of a morphine-like substrate in the CNS. In the early 1980s, the purification of β-endorphin and enkephalin implicated these opiates as key players in acupuncture in humans and animals (Clement-Jones et al., 1980; Pert et al., 1981; Kiser et al., 1983). The research revealed that acupuncture increased levels of plasma enkephalin and cerebrospinal fluid β-endorphin in humans after acupuncture treatment.

Investigations into the relationship between different levels of EA analgesia have found that met-enkephalin, β-endorphin, and endomorphin are preferably stimulated by low-frequency (2 Hz) EA, while dynorphin is the only opioid peptide that responds to high-frequency (100 Hz) EA stimulation (Han, 2004). Further experimental research has shown that a dense-and-disperse (DD) mode of EA stimulation alternating 2 Hz with 100 Hz, each lasting for 3 s, evokes the simultaneous release of both enkephalins and dynorphins, resulting in a synergistic interaction that maximizes the therapeutic effect (Han, 2004). These findings have been substantiated in clinical research (Han, 2011).

### Interference With the Central Sensitization Process

The general consensus among scientists in the pain research field is that chronic pain states in humans are driven by three core mechanisms: nociceptive and neuropathic pain mechanisms, as well as central sensitization (Harte et al., 2018). The term central sensitization conveys the understanding that CNS mechanisms are implicated in the amplification of pain, independently of peripheral injury or inflammation (Harte et al., 2018). Effective pain management relies upon the recognition of central sensitization and whether it is resulting from ongoing nociceptive input or is occurring despite no obvious peripheral driver (Harte et al., 2018).

The analgesic effects of manual acupuncture and EA are produced through multiple pathways and interfere with the central sensitization process by reducing levels of inflammatory mediators in the peripheral tissue, including substance P, interleukin (IL)-1β, IL-8, IL-10, and tumor necrosis factor (TNF)-α (Zhang et al., 2012; Lai et al., 2019). Possible processes whereby acupuncture reduces central sensitization include the release of the endogenous opioid, adrenergic, and 5-hydroxytryptamine (5-HT, serotonin) receptors, the *N*-methyl-D-aspartate/α-amino-3-hydroxy-5-methyl-4-isozazolepropionin acid/kainate (NMDA/AMPA/KA) pathways, and afferent segmental inhibition (Lai et al., 2019). Segmental acupuncture analgesia postulates that high-frequency, low-intensity EA activates myelinated afferent A-fibers that reduce nociceptive transmission via inhibitory interneurons in the spinal dorsal root, reducing the response to painful stimuli within the same spinal segment (Baeumler et al., 2014, 2019).

### The Role of the Adenosine A_1_ Receptor

Researchers have noted that the hypothesized activation of centrally acting, endogenous opioid peptides in the CNS in response to acupuncture stimulation cannot completely explain why acupuncture needling is conventionally applied to an acupoint adjacent to the area of pain (Goldman et al., 2010). In mouse models of inflammatory pain, Goldman et al. (2010) found that analgesic effects from manual acupuncture are mediated by the release of the transmitter adenosine. Injecting the mice after acupuncture treatment with deoxycoformycin, an adenosine deaminase inhibitor, prolonged the accumulation of adenosine and the antinociceptive effects of acupuncture. The researchers suggest that activation of the adenosine A_1_ receptor is necessary for acupuncture analgesia (Goldman et al., 2010).

### Non-opioid Mechanisms

Conversely, other research using human subjects failed to demonstrate naloxone reversal of EA analgesia, calling into question the premise that endorphins play a significant role in acupuncture analgesia (Chapman et al., 1983). In that study, 14 healthy adult volunteers who demonstrated EA analgesia during electrical stimulation of the LI4 acupoint were randomly assigned to naloxone 1.2 mg or normal saline (Chapman et al., 1983). Naloxone failed to reverse pain thresholds elevated by acupuncture, suggesting that other mechanisms are involved in acupuncture-induced analgesia (Chapman et al., 1983).

## The Role of the Endocannabinoid System (ECS) in Electroacupuncture (EA) Analgesia

Greater understanding of the ECS has encouraged investigations into the role of the ECS in acupuncture analgesia. This research is discussed below.

### EA-Induced Analgesia and Anti-inflammatory Effects Depend on CB_1_ Receptors and/or CB_2_ Receptors

EA analgesia in inflammatory pain relates to the dopamine system and CB_1_ receptors in the striatum (Shou et al., 2013). In the complete Freund’s adjuvant (CFA)-induced model of arthritis in mice, EA significantly increased CB_1_ receptor expression in the striatum and prolonged paw withdrawal latency; both effects were attenuated by the CB_1_ receptor antagonist AM251, indicating an important role for the CB_1_ receptor in EA-induced analgesia (Shou et al., 2013). The study researchers also reported that EA upregulated dopamine D_1_ and D_2_ receptor mRNA expression in the corpus striatum, which was effectively blocked by AM251 (Shou et al., 2013). They suggested that EA analgesia in inflammatory pain is due to cross-modulation between the dopamine system and CB_1_ in the striatum (Shou et al., 2013).

CB_1_ and CB_2_ receptors contribute to antinociceptive and anti-inflammatory effects of EA in arthritis of the rat temporomandibular joint. In one study, researchers found that EA significantly inhibited mechanical hypernociception in rats with acute arthritis induced by zymosan in the temporomandibular joint (TMJ) (Gondim et al., 2012). This effect was reversed by AM251, although AM251 failed to affect the EA-induced anti-inflammatory response in the TMJ (Gondim et al., 2012). In contrast, the CB_2_ receptor antagonist AM630 reversed the EA-induced anti-inflammatory effect but did not alter its antinociceptive effect (Gondim et al., 2012). These findings are supported by another study implicating the involvement of CB_1_ receptors in EA-induced orofacial antinociception among rats exposed to noxious facial heat (Almeida et al., 2016). In this experimental study, EA alone at acupoint ST36 induced antinociception that was blocked by pretreatment with AM251, but not by AM630 (Almeida et al., 2016). Moreover, the antinociceptive effects of EA were prolonged and intensified by pretreatment with an endocannabinoid metabolizing enzyme inhibitor (MAFP) or an anandamide reuptake inhibitor (VDM11) (Almeida et al., 2016). Acupuncture at ST36 (located below the knee) is generally used to treat dysfunctional gastrointestinal activity (Cheong et al., 2013) and rarely for diseases of the head and mouth (Yang Y. et al., 2012). However, the study by Almeida et al. (2016) revealed that ST36 is also related to the ECS.

### CB_1_ Receptors in the vlPAG Are Critically Important for the Mechanisms of the EA Antinociceptive Effect

The vlPAG midbrain region is an important site of action in cannabinoid-induced antinociception and a likely supraspinal site of orexin antinociception (Ho et al., 2011). Orexin A and B are hypothalamic peptides that activate postsynaptic orexin 1 and orexin 2 receptors (OX_1_Rs and OX_2_Rs), which have many roles in physiological processes including energy homeostasis, stress processing, visceral functions, reward seeking behavior, cognition, endocrine functions, arousal, and pain modulation (Razavi and Hosseinzadeh, 2017). OX_1_Rs and OX_2_Rs are densely distributed in the PAG (Ho et al., 2011). In particular, orexin A can induce an opioid-independent analgesic mechanism that is mediated by OX_1_R-initiated 2-AG signaling in the vlPAG (Ho et al., 2011; Chen et al., 2018). Subsequent research using murine pain models has demonstrated how low-frequency median nerve stimulation (MNS) using acupuncture needles at the PC6 acupoint (EA-PC6) induces analgesia involving both CB_1_ receptors and OX_1_Rs (Chen et al., 2018). MNS-PC6-induced antinociception (a procedure equivalent to EA-PC6) was prevented by systemic blockade of OX_1_Rs or CB_1_ receptors, but not by opioid receptor antagonists (Chen et al., 2018). Systemic blockade of OX_1_Rs or CB_1_ receptors also prevented the EA-PC6-induced reduction in GABA levels in the vlPAG microdialysate (Chen et al., 2018). Notably, EA-PC6-induced analgesia was reduced by intra-vlPAG inhibition of 2-AG synthesis, implicating a cannabinoid (2-AG)-dependent mechanism, which was supported by the finding that EA-PC6-induced antinociception was markedly attenuated in *Cnr1^–/–^* mice, which lack the CB_1_ receptor (Chen et al., 2018). These findings suggest that PC6-targeting low-frequency MNS activates hypothalamic orexin neurons, releasing orexins that activate postsynaptic OX_1_Rs in the vlPAG to generate 2-AG, which inhibits GABA release through CB_1_ receptors in the vlPAG and induces analgesia by disinhibiting vlPAG outputs (Chen et al., 2018). These findings have been reinforced in further investigations by these researchers, who report that repeated EA-PC6 treatments remained fully effective in morphine-tolerant mice with neuropathic pain caused by chronic constriction injury (CCI) of the sciatic nerve, via a mechanism involving OX_1_Rs and CB_1_ receptors (Lee et al., 2020).

In other research involving mice with knee osteoarthritis, EA increased the levels of CB_1_ receptors and 2-AG in the vlPAG to reduce chronic knee osteoarthritis pain; the effect of EA on pain hypersensitivity was reversed when AM251 was injected into the vlPAG (Yuan et al., 2018b). Subsequent research by the same group discovered that knocking out the CB_1_ receptor on GABAergic neurons abolished most of the antinociceptive effects of EA in mice with CCI or knee osteoarthritis, while knocking out the CB_1_ receptor on glutamatergic neurons in the vlPAG only partly reduced the effects of EA (Zhu et al., 2019). The researchers proposed that inhibition of GABAergic neurons and activation of glutamatergic neurons in the vlPAG through CB_1_ receptors contribute to EA-induced analgesia (Zhu et al., 2019).

Counterintuitively, while some research has shown that low doses of μ-opioid receptor antagonists levonaloxone, naltrexone, cyclazocine, and diprenorphine block EA analgesia (Cheng and Pomeranz, 1980), other investigations have demonstrated that ultra-low-dose naltrexone (0.001–0.004 mg/day) enhances opiate-induced analgesia while also reducing opioid tolerance and dependence in chronic pain conditions (Chindalore et al., 2005; Webster, 2007). Some researchers have hypothesized that low-dose naltrexone (3–4.5 mg/day) could synergistically enhance acupuncture-induced pain relief (Hesselink and Kopsky, 2011). In support of this hypothesis, low-dose naltrexone appears to exert hypoalgesic effects in healthy human volunteers exposed to noxious electrocutaneous stimulation (France et al., 2007), blocks acute tolerance to morphine and attenuates morphine-elicited thermal hyperalgesia in rats (McNaull et al., 2007), and enhances cannabinoid-induced antinociception in rats (Paquette and Olmstead, 2005). Preclinical research has effectively illustrated interactions between the cannabinoid system and opioid antinociception, showing that AM251 can completely block morphine-induced peripheral and central antinociception in a rat model of inflammatory pain and in the tail-flick test in mice, while AM630 is only partially effective or fails to antagonize antinociception induced by morphine (Pacheco et al., 2008, 2009). These researchers have therefore suggested that μ-opioid-induced antinociception involves peripheral (Pacheco et al., 2008) or central (Pacheco et al., 2009) activation of CB_1_ and/or CB_2_ receptors that potentiate the opioid-induced antinociceptive mechanisms. Other evidence suggests that a regimen of low-dose THC combined with low-dose morphine enhances opioid analgesic potency without increasing undesirable side effects (Cichewicz, 2004). Further support for considerable crosstalk between the opioid and endocannabinoid pathways comes from studies involving transgenic mice with inactivated CB_1_ or CB_2_ receptors [*cnr1* and *cnr2* knockout (KO) mice], which exhibit marked reductions in peripheral or spinal morphine analgesia (Desroches et al., 2014). This loss in morphine analgesia was not explained by downregulation in μ-opioid spinal expression nor by altered binding properties or G protein coupling of the μ-opioid receptor in *cnr1*KO and *cnr2*KO mice (Desroches et al., 2014).

### CB_2_ Receptors in the Peripheral Tissue Mediate the Anti-inflammatory Effects of Acupuncture

It is possible that the roles of CB_1_ and CB_2_ receptors differ according to the type of pain condition. For instance, EA significantly increased levels of AEA in inflamed skin tissue and produced antinociceptive effects by activating peripheral CB_2_ receptors in a rat model of CFA-induced inflammatory pain (Chen et al., 2009), while CB_1_ receptors appeared to mediate the anti-inflammatory effect of EA in a rat model of migraine (Zhang et al., 2016). In the CFA model of inflammatory pain induced by the local injection of CFA into the hind paw of rats, local pretreatment with AM630 significantly attenuated the antinociceptive effect of EA, whereas local pretreatment with AM251 had no significant effect on EA analgesia (Chen et al., 2009). In a follow-up study using the same inflammatory pain model, the researchers found increased numbers of CB_2_ receptors on macrophages, T-lymphocytes, and keratinocytes in the epidermis and dermis in response to CFA injections; further increases were observed in all of these cells expressing CB_2_ receptors in the inflamed skin of rats in the CFA plus 2 and 100 Hz EA groups (Zhang et al., 2010). CFA plus 2 Hz EA and CFA plus 100 Hz EA also significantly increased mRNA and protein levels of CB_2_ receptors in the inflamed skin tissue (Zhang et al., 2010). In another study involving rats with CFA-induced inflammatory pain, EA appeared to reduce pain by inhibiting activation of the NLRP3 inflammasome in inflamed skin tissue through CB_2_ receptor stimulation (Gao et al., 2018). Similarly, other researchers found that EA-induced activation of the CB_2_ receptor inhibited chronic pain in mice with knee osteoarthritis (Yuan et al., 2018a). In their study, EA treatment was associated with significant increases in levels of CB_2_ receptor expression in fibroblasts and significant reductions in IL-1β-positive cells in the knee meniscus; knockout of the CB_2_ receptor blocked EA analgesia and EA had no effect upon IL-1β expression in *CB2^–/–^* mice (Yuan et al., 2018a).

EA analgesia in inflammatory pain may also be due to an interaction between peripheral CB_2_ receptors and endogenous opioids (Su et al., 2011). β-Endorphin, the endogenous ligand of the opioid peptide, is derived from the precursor proopiomelanocortin (POMC). In a rat model of inflammatory pain, mRNA levels of POMC and protein levels of β-endorphin were significantly increased in inflamed skin tissues after rats were treated with the CB_2_ receptor agonist AM1241 or EA, while AM630 significantly reduced these effects (Su et al., 2011). Percentages of β-endorphin-immunoreactive keratinocytes, macrophages, and T-lymphocytes were also significantly increased by AM1241 or EA, whereas pretreatment with AM630 blocked these effects (Su et al., 2011). Thus, a peripheral interaction between μ-opioid receptors and CB_2_ receptors is implicated in EA analgesia in inflammatory pain.

The same group of researchers subsequently reported that EA appears to reduce inflammatory pain and proinflammatory cytokine expression by activating CB_2_ receptors in CFA-induced skin inflammation (Su et al., 2012). EA at GB30 and GB34 acupoints and also local injections of AM1241 significantly decreased thermal hyperalgesia and mechanical allodynia in inflammatory skin tissue; the antinociceptive effect of EA was blocked by pretreatment with AM630 (Su et al., 2012). EA or AM1241 treatment also significantly reduced IL-1β, IL-6, and TNF-α mRNA and protein levels in inflamed skin tissue; these inhibitory effects were reversed by AM630 pretreatment (Su et al., 2012).

The placebo effect in acupuncture analgesia is well-recognized (Musial, 2019). The ECS can also play an important role in placebo analgesia. Research has revealed the secretion of endogenous endocannabinoids from the brain during a placebo response to pain, when non-opioid drugs were used in the preconditioning phase (Benedetti, 2012). For instance, the specific CB_1_ receptor antagonist rimonabant can effectively block placebo analgesia elicited by non-opioid pharmacological conditioning with non-steroidal anti-inflammatory drugs (NSAIDs) in healthy volunteers (Benedetti et al., 2011). More animal and human studies are needed to determine how CB_1_ receptors contribute to placebo and acupuncture analgesia.

Publications from 2009 onward supporting the involvement of endocannabinoids in EA analgesia are summarized in Table 1.

**TABLE 1:** Reports published between 2009 and 2020 (cited in ascending date order) describing the involvement of the ECS in EA analgesia.

Pain model	Acupuncture treatment	Major findings	Conclusions	References
Inflammatory pain/CFA injection	2 or 100 Hz EA (1 mA, 30 min) at GB30 and GB34, on days 2, 4, and 6 after CFA injection	• EA at 2 and 100 Hz significantly reduced thermal hyperalgesia and mechanical allodynia associated with CFA injections. • EA significantly increased endogenous levels of AEA in inflamed skin tissue. • EA-induced antinociception was significantly reduced by AM630, but not significantly altered by AM251. AM251 and AM630 were each given as subcutaneous injections into the dorsal surface of the left hind paw 5 min prior to sham EA or EA treatment.	EA appears to enhance the local release of endogenous AEA from inflamed skin tissue. EA analgesia in inflammatory pain involves the activation of peripheral CB_2_ receptors.	Chen et al., 2009
Inflammatory pain/CFA injection	2 or 100 Hz (1 mA, 30 min) at GB30 and GB34 once every other day, starting from the second day after CFA injection, for 7 days	• EA at 2 and 100 Hz significantly increased CB_2_R mRNA and protein expression in inflamed skin tissue. • EA 2 and 100 Hz significantly increased quantities of CB_2_R-immunoreactive keratinocytes, macrophages, and T-lymphocytes in inflamed skin tissue.	EA upregulates CB_2_ receptor expression in keratinocytes and inflammatory cells in inflamed skin tissue.	Zhang et al., 2010
Inflammatory pain/CFA injection	2 Hz EA (1 mA, 30 min) at GB30 and GB34, on days 2, 4, and 6 after CFA injection	• EA or AM1241 significantly reduced thermal hyperalgesia and mechanical allodynia; pretreatment with β-funaltrexamine (a selective μ-opioid receptor antagonist) attenuated these effects. • EA or AM1241 significantly increased POMC mRNA and β-endorphin protein levels in inflamed skin tissues; these effects were significantly reduced by pretreatment with AM630. AM1241 or AM630 was injected subcutaneously into the dorsal surface of the left hind paw 5 min before each session of sham EA or EA treatment. • EA also significantly increased the percentage of β-endorphin-immunoreactive keratinocytes, macrophages, and T-lymphocytes in inflamed skin tissue; AM630 prevented these effects.	EA increases endogenous opioid expression in keratinocytes and infiltrating immune cells at the inflammatory site by activating peripheral CB_2_ receptors.	Su et al., 2011
Inflammatory pain/CFA injection	2 Hz EA (1 mA, 30 min) at GB30 and GB34, on days 2, 4, and 6 after CFA injection	• EA or AM1241 treatment significantly reduced thermal hyperalgesia and mechanical allodynia after CFA injection. • AM630 significantly attenuated EA antinociception. AM1241 or AM630 was injected subcutaneously into the dorsal surface of the left hind paw 5 min before each session of sham EA or EA treatment. • EA or AM1241 treatment significantly reduced IL-1β, IL-6, and TNF-α mRNA and protein levels in inflamed skin tissue. • The inhibitory effects of EA on these cytokines were significantly reversed by pretreatment with AM630.	EA activation of CB_2_ receptors reduces inflammatory pain and proinflammatory cytokine expression in inflamed tissue.	Su et al., 2012
Inflammatory pain/zymosan administration in the TMJ	10 Hz EA (3 mA, 30 min) at LI4, LI11, ST36 and ST34, 1 h before or 2 h after zymosan administration in the TMJ	• EA significantly inhibited zymosan-induced hypernociception. • EA antinociception was significantly reversed by AM251. • EA anti-inflammatory effects were reversed by AM630. Both AM251 and AM630 were given as single, IP injections 10 min prior to EA treatment. • *CB_1_R* and *CB_2_R* gene expression was upregulated 6 h after zymosan-induced arthritis in EA-treated rats. • In EA-treated rats, *CB_1_R* gene expression was significantly increased at 6 h after zymosan administration and increased still further at 24 h, whereas *CB_2_R* gene expression peaked at 6 h after zymosan administration and was downregulated at 24 h.	Antinociceptive and anti-inflammatory effects of EA appeared to be mediated through CB_1_ and CB_2_ receptor activation.	Gondim et al., 2012
Inflammatory pain/CFA injection	2/100 Hz EA (1.0, 2.0, 3.0 mA, 20 min) at ST36 and BL60, once every other day starting from the 4th day after CFA injection, for 4 sessions	• EA improved thermal hyperalgesia and significantly increased levels of CB_1_ expression in rat striatum. • AM251 significantly attenuated EA-induced increases in CB_1_ expression. Single, IP injections of AM251 were administered on study day 10. • EA upregulated dopamine D_1_ and D_2_ receptor mRNA expression in the corpus striatum, which was effectively blocked by AM251.	EA analgesia in inflammatory pain is associated with upregulation of dopamine and CB_1_ receptors in the rat corpus striatum.	Shou et al., 2013
Migraine (induced by electrical stimulation of the trigeminal ganglion; TGES)	Ipsilateral 2/15 Hz EA (1 mA, 30 min) at GB20 and TE5 once daily for 5 days before TGES	• EA significantly attenuated TGES-induced increases in serum CGRP and PGE_2_ levels, and inhibited the TGES-induced increase in neurogenic PPE. • EA significantly attenuated TGES-induced increases in COX2 and IL-1β protein levels in the trigeminal ganglion. • The effects of EA were reversed by CB_1_R antagonism.	CB_1_ receptors appeared to mediate EA anti-inflammatory effects in a rat model of migraine.	Zhang et al., 2016
Orofacial pain (induced by noxious heat applied to the face)	100 Hz EA (0.5 mA, 20 min) at ST36	• EA-induced antinociception was prolonged and intensified by pretreatment with an endocannabinoid metabolizing enzyme inhibitor (MAFP) and an anandamide reuptake inhibitor (VDM11). • EA-induced antinociception was blocked by pretreatment with AM251, but not by AM630. All study drugs were injected as single, IP doses, 10 min prior to EA treatment.	EA orofacial antinociception appeared to involve activation of CB_1_ receptors.	Almeida et al., 2016
Inflammatory pain/MIA injection	*Knee OA model*: 2, 15, or 100 Hz EA (1 mA, 30 min) at Ex-LE4 and ST35, starting from 2 days after IA-injected MIA, once every other day for 4 weeks	• Chronic pain-induced reductions in levels of CB_1_Rs and 2-AG expression in the midbrain were reversed by EA treatment. • Microinjection of AM251 into the vlPAG reversed the effects of EA on pain hypersensitivity and DNIC function. • In *GABA-CB1^–/–^* mice subjected to knee OA induction, the reduced thermal latencies and tactile thresholds were not significantly affected by EA. Similarly, EA had no effect on the reduction in 5-HT levels in the medulla following the induction of knee OA in *GABA-CB1^–/–^* mice.	It appears that the 2-AG-CB_1_R-GABA-5-HT signaling pathway underlies the effects of EA on descending inhibitory control of 5-HT, improving DNIC function and inhibiting chronic pain. CB_1_Rs on GABAergic neurons were involved in the effects of EA on DNIC function and descending inhibitory control of 5-HT in the medulla.	Yuan et al., 2018b
Inflammatory pain/MIA injection	*Knee OA model*: 2 Hz EA (1 mA, 30 min) at Ex-LE4 and ST35, starting from 2 days after IA-injected MIA, once every other day for 4 weeks	• EA significantly increased levels of CB_2_R expression in fibroblasts and significantly reduced IL-1β-positive cells in the knee meniscus. • Knockout of the CB_2_R blocked EA analgesia.	EA reduced levels of IL-1β expression by activating CB_2_ receptors, which effectively reduced chronic pain in mice with knee OA.	Yuan et al., 2018a
Heat hyperalgesia and neuropathic pain	*Hot-plate test*: 2 Hz EA-PC6 (2 mA, for 20 min) *CCI model*: EA-PC6, non-MNS, or sham-PC6 were applied on postoperative day 8	• EA-PC6 reduced acute thermal nociceptive responses and neuropathy-induced mechanical allodynia; these effects were prevented by systemic or intra-vlPAG injection of an antagonist of OX_1_Rs or CB_1_Rs, but not by opioid receptor antagonists. • EA-PC6 increased the number of c-Fos-immunoreactive hypothalamic orexin neurons, and led to higher orexin A and lower GABA levels in the vlPAG. • EA-PC6-induced nociception was prevented by intra-vlPAG inhibition of 2-AG synthesis and was attenuated in *Cnr1^–/–^* mice.	EA-PC6 induces the release of an endogenous neuropeptide (orexin) from the hypothalamus to inhibit pain responses in mice through a CB_1_R-dependent cascade that reduces inhibitory GABAergic control in the vlPAG.	Chen et al., 2018
Inflammatory pain/CFA injection	QD 2 Hz EA (1 mA, 30 min) at GB30 and GB34 on days 2, 4, and 6 after CFA injections	• EA significantly reduced CFA-induced thermal hyperalgesia and mechanical allodynia and attenuated CFA-induced activation of the NLRP3 inflammasome in inflamed skin tissues. • *In vitro* studies in a rat alveolar macrophage cell line revealed that activation of CB_2_ receptors inhibited NLRP3 inflammasome activation.	EA appears to relieve inflammatory pain by inhibiting NLRP3 inflammasome activation in inflamed skin tissues through CB_2_ receptors.	Gao et al., 2018
Inflammatory and neuropathic pain	*CCI model:* QD 2 Hz EA (1 mA, 30 min) at GB30 and GB34, starting from the 8th postoperative day and ending on the 14th postoperative day	*Knee OA model:* QD 2 Hz EA (1 mA, 30 min) at Ex-LE4 and ST35, starting from the 15th day after IA-injected MIA and ending on the 21st day *Sham:* Acupuncture needles were inserted into the study acupoints without electrical stimulation or manual manipulation	• Chemogenetic inhibition of GABAergic neurons in the vlPAG mimicked the effects of EA. • The combination of chemogenetic activation of GABAergic neurons and chemogenetic inhibition of glutamatergic neurons in the vlPAG was needed to reverse the effects of EA. • Specifically knocking out CB_1_Rs on GABAergic neurons in the vlPAG abolished the EA effect on pain hypersensitivity.	EA synchronously inhibits GABAergic neurons and activates glutamatergic neurons in the vlPAG through CB_1_Rs to produce EA-induced analgesia. The CB_1_Rs on GABAergic neurons localized in the vlPAG was the basis of the EA effect on pain hypersensitivity.	Zhu et al., 2019

*2-AG, 2-arachidonoylglycerol; 5-HT, 5-hydroxytryptamine or serotonin; AM1421, CB_2_ receptor agonist; AM251, CB_1_ receptor antagonist; AM630, CB_2_ receptor antagonist; BID, twice daily; BL60, Kunlun; CB_1_R, cannabinoid 1 receptor; CCI, chronic constriction nerve injury; CFA, complete Freund’s adjuvant; CGRP, calcitonin gene-related peptide; DNIC, diffuse noxious inhibitory control; EA, electroacupuncture; ECS, endocannabinoid system; Ex-LE4, Neixiyan; GB30, Huantiao; GABA, γ-aminobutyric acid; GB20, Fengchi; GB34, Yanglingquan; IA, intra-articular; IP, intraperitoneal; IT, intrathecal; LI4, Hegu; LI11, Quchi; MIA, monosodium iodoacetate; MNS, median nerve stimulation; non-MNS, the same electrical stimulation as in the MNS group via acupuncture needles inserted in the middle of the lateral deltoid muscle (a non-median nerve-innervated location); OA, osteoarthritis; OX_1_R, orexin 1 receptor; PC6, Neiguan; PPE, plasma protein extravasation; QD, once daily; p-ERK1/2, phosphorylated-extracellular signal-regulated kinase 1/2; PGE_2_, prostaglandin E_2_; sham-PC6, bilateral needle insertion at the PC6 acupoint without electrical stimulation; ST34, Liangqiu; ST35, Dubi; ST36, Zusanli; TE5, Waiguan; TGES, electrical stimulation of the trigeminal ganglion; vlPAG, ventrolateral periaqueductal gray.*

### Functions of the Acupoints Selected in the Reviewed Studies

The acupoints reviewed in this article and listed in Table 1 are primarily found in the upper and lower limbs. GB30 (*Huantiao*) is located on the sciatic nerve path and is used in TCM as a basic point in low back pain (Shao et al., 2015) and for motor function treatment (Yeo et al., 2014). GB34 (*Yanglingquan*) is found on the common peroneal nerve and is used as an additional point to GB30 when low back pain is associated with lower extremity numbness and pain (Shao et al., 2015). Stimulation of GB34 activates the prefrontal cortex, the precentral gyrus and putamen in patients with Parkinson’s disease; areas of the brain that exhibit dysfunction due to nigral dopamine depletion (Yeo et al., 2014). LI4 (*Hegu*) is located on the dorsum of the hand and LI11 (*Quchi*) at the elbow; both are suggested to be particularly useful for improving neck-shoulder-arm disorders (Shiro et al., 2014), with clinical evidence describing the relief of stress, facial pain, headache, toothache, neck, and shoulder pain (LI4) (He et al., 2004; Shen et al., 2009; Pavão et al., 2010; Grillo et al., 2014; Wang et al., 2015), pain-related conditions, and common fever (LI11) (Choi et al., 2018). Acupoints ST34 (*Liangqiu*, above the laterosuperior border of the patella), ST35 (*Dubi*, at the lower border of the patellar) and ST36 (*Zusanli*, on the anterior of the leg lateral to the edge of the tibia, below ST35) are effective for postoperative pain control (Liu et al., 2015); stimulation of ST36 is frequently used to treat dyskinesia and facilitate motor recovery after stroke, to treat pain, hypertension, and other physiological dysfunctions (Sun et al., 2019) such as migraine (Zhao et al., 2012). The acupoint combination of GB20 (*Fengchi*, situated below the base of the skull) and TE5 (*Waiguan*, near the dorsal wrist crease between the radius and ulna) is frequently used for treating migraine (Zhao et al., 2012), while EX-LE4 (*Neixiyan*) and ST35 (both located on the knee) are frequently paired for pain relief, such as in knee osteoarthritis (Ng et al., 2003). PC6 (*Neiguan*, on the palm side of the wrist and located on the median nerve path) is a classical acupoint that is used to treat cardiovascular disorders (Li et al., 2012), for providing postoperative analgesia (Xie et al., 2014), and for preventing nausea and vomiting (Cheong et al., 2013), while BL60 (*Kunlun*, on the posterior aspect of the knee) is frequently used in acupoint combinations for the treatment of low back pain (Lee et al., 2013).

## Conclusion

No single mechanism can explain EA analgesia. Early preclinical investigations demonstrated that naloxone antagonizes EA analgesia, suggesting that the analgesic action is related to the release of a morphine-like factor in the CNS, supporting the endorphin hypothesis of acupuncture analgesia (Pomeranz and Chiu, 1976). Subsequent research revealed elevated levels of β-endorphin in human cerebrospinal fluid after acupuncture for recurrent pain (Clement-Jones et al., 1980) and in plasma met-enkephalin after EA in patients with chronic pain (Kiser et al., 1983). Different frequencies of EA have been found to elucidate different opiate secretions; low-frequency EA (2 Hz) is associated with analgesia involving met-enkephalin, β-endorphin, and endomorphin, which activate the μ- and δ-opioid receptors, while high-frequency EA (100 Hz) stimulates dynorphin, which activates the κ-opioid receptor (Han, 2004). Evidence on EA analgesia also implicates the midbrain monoamines serotonin and norepinephrine, and anti-inflammatory mechanisms mediated by opioid and non-opioid receptors in the periphery (Zhang et al., 2014).

The discovery of the cannabinoid receptors and endocannabinoids has inspired many investigations that have targeted ECS proteins, the cannabinoid receptors, and the enzymes responsible for the biosynthesis and degradation of the endogenous cannabinoid receptor ligands, in the hope of discovering therapeutic targets that can be treated with novel drugs for a wide range of diseases, including pain (Stasiulewicz et al., 2020). However, these investigations have been complicated by adverse effects arising from the multidirectional nature of the ECS and its inter-relationships with other pharmacological systems and biochemical pathways (Stasiulewicz et al., 2020).

**SCHEME 1:**
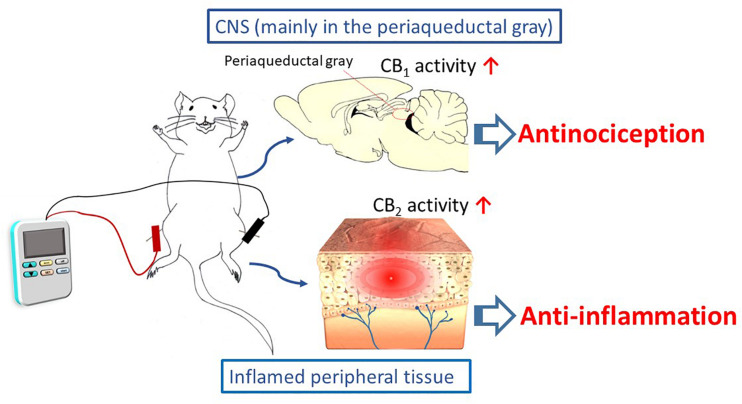
The cannabinoid pathway, including the CB_1_ and CB_2_ receptors, mediates the analgesic and anti-inflammatory effects of electroacupuncture.

EA interventions are potentially appropriate for activating the ECS. The evidence discussed in this review suggests that EA inhibits inflammatory and neuropathic pain, and that these effects may be associated with modulations of cannabinoid signaling within the ECS. This signaling is illustrated in Scheme 1. It appears that the cannabinoid pathway mediates the analgesic and anti-inflammatory effects of acupuncture, via the CB_1_ and CB_2_ receptors, respectively. However, the current results are all generated from animal studies. Evidence from animal studies remains fragmentary and clinical evidence is lacking. In contrast, evidence for the endorphins theory is supported by not only animal studies, but also human investigations.

Clearly, EA increases different types of endogenous opioids in humans, while naloxone, an opioid receptor antagonist, appears to attenuate acupuncture analgesia in humans. To fully inform the ECS theory of EA analgesia, further human studies are called for. As with the evidence in support of the endorphin hypothesis, biochemical evidence such as measurements of cerebrospinal fluid (CSF) concentrations of endocannabinoids are needed to show how the ECS is influenced by acupuncture interventions. For instance, one study has identified significantly lower CSF concentrations of AEA in patients with chronic migraine and those with probable chronic migraine and probable analgesic-overuse headache compared with non-migraine controls (Sarchielli et al., 2007). Interestingly, CB_1_ receptor antagonism is a promising strategy in the treatment of obesity. However, CNS side effects associated with rimonabant and other CB_1_ receptor antagonists emphasized the need for new classes of peripherally acting CB_1_ receptor antagonists that do not affect the intricate balance between central and peripheral physiological signaling (Sharma et al., 2015; Lv et al., 2019). Ongoing efforts to develop safer selective peripherally acting CB_1_ receptor antagonists, potent novel CB_1_ receptor antagonists or inverse agonists may eventually result in therapeutics that target CB_1_ receptors with reduced CNS side effects (Sharma et al., 2015; Yadav and Murumkar, 2018; Khan N. et al., 2019; Micale et al., 2019). At that point, it would be interesting to determine whether or not CB_1_ receptor antagonists attenuate EA analgesia in humans. We would then expect to have much more clarity around the biological basis underlying the mechanisms of EA-induced antinociception involving CB_1_ and CB_2_ receptors in the ECS.

## Author Contributions

Y-HC conceived and advised on the manuscript. IM wrote the first draft of the manuscript. Both authors revised the manuscript and agreed with the published version of the manuscript.

## Conflict of Interest

The authors declare that the research was conducted in the absence of any commercial or financial relationships that could be construed as a potential conflict of interest.
